# Supplementary material for: Smooth Interpolating Curves with Local Control and Monotone Alternating Curvature
Source: Comput Graph Forum. 2022 Oct 6;41(5):25–38. doi: 10.1111/cgf.14600 (PMC9827861; doi:10.1111/cgf.14600)
Supplement: Supplementary file 1 — Supplement Material [file CGF-41-25-s001.zip › Local-Smooth-Interpolating-MonoCurvature/extern/clothoids/docs/api-cpp/program_listing_file_Clothoids_Triangle2D.hxx.html]

Program Listing for File Triangle2D.hxx — Clothoids v2.0.9

### Navigation

- index
- toc
- Clothoids »
- Program Listing for File Triangle2D.hxx

# Program Listing for File Triangle2D.hxx¶

↰ Return to documentation for file (`Clothoids/Triangle2D.hxx`)

```
/*--------------------------------------------------------------------------*\
 |                                                                          |
 |  Copyright (C) 2017                                                      |
 |                                                                          |
 |         , __                 , __                                        |
 |        /|/  \               /|/  \                                       |
 |         | __/ _   ,_         | __/ _   ,_                                |
 |         |   \|/  /  |  |   | |   \|/  /  |  |   |                        |
 |         |(__/|__/   |_/ \_/|/|(__/|__/   |_/ \_/|/                       |
 |                           /|                   /|                        |
 |                           \|                   \|                        |
 |                                                                          |
 |      Enrico Bertolazzi                                                   |
 |      Dipartimento di Ingegneria Industriale                              |
 |      Universita` degli Studi di Trento                                   |
 |      email: enrico.bertolazzi@unitn.it                                   |
 |                                                                          |
\*--------------------------------------------------------------------------*/


namespace G2lib {

  /*\
   |   _____     _                   _      ____  ____
   |  |_   _| __(_) __ _ _ __   __ _| | ___|___ \|  _ \
   |    | || '__| |/ _` | '_ \ / _` | |/ _ \ __) | | | |
   |    | || |  | | (_| | | | | (_| | |  __// __/| |_| |
   |    |_||_|  |_|\__,_|_| |_|\__, |_|\___|_____|____/
   |                           |___/
  \*/
  class Triangle2D {
    real_type m_p1[2], m_p2[2], m_p3[2];
    real_type m_s0;
    real_type m_s1;
    int_type  m_icurve;

  public:

    Triangle2D( Triangle2D const & t )
    { *this = t; }

    Triangle2D( ) {
      m_p1[0]  = m_p1[1] = m_p2[0] = m_p2[1] = m_p3[0] = m_p3[1] = 0;
      m_s0     = 0;
      m_s1     = 0;
      m_icurve = 0;
    }

    Triangle2D(
      real_type x1, real_type y1,
      real_type x2, real_type y2,
      real_type x3, real_type y3,
      real_type s0, real_type s1,
      int_type  icurve
    ) {
      m_p1[0]  = x1; m_p1[1] = y1;
      m_p2[0]  = x2; m_p2[1] = y2;
      m_p3[0]  = x3; m_p3[1] = y3;
      m_s0     = s0;
      m_s1     = s1;
      m_icurve = icurve;
    }

    Triangle2D(
      real_type const p1[2],
      real_type const p2[2],
      real_type const p3[2],
      real_type       s0,
      real_type       s1,
      int_type        icurve
    ) {
      m_p1[0] = p1[0]; m_p1[1] = p1[1];
      m_p2[0] = p2[0]; m_p2[1] = p2[1];
      m_p3[0] = p3[0]; m_p3[1] = p3[1];
      m_s0     = s0;
      m_s1     = s1;
      m_icurve = icurve;
    }

    ~Triangle2D() {}

    Triangle2D const &
    operator = ( Triangle2D const & t ) {
      m_p1[0] = t.m_p1[0]; m_p1[1] = t.m_p1[1];
      m_p2[0] = t.m_p2[0]; m_p2[1] = t.m_p2[1];
      m_p3[0] = t.m_p3[0]; m_p3[1] = t.m_p3[1];
      m_s0     = t.m_s0;
      m_s1     = t.m_s1;
      m_icurve = t.m_icurve;
      return *this;
    }

    void
    build(
      real_type const p1[2],
      real_type const p2[2],
      real_type const p3[2],
      real_type       s0,
      real_type       s1,
      int_type        icurve
    ) {
      m_p1[0] = p1[0]; m_p1[1] = p1[1];
      m_p2[0] = p2[0]; m_p2[1] = p2[1];
      m_p3[0] = p3[0]; m_p3[1] = p3[1];
      m_s0     = s0;
      m_s1     = s1;
      m_icurve = icurve;
    }

    void
    build(
      real_type x1, real_type y1,
      real_type x2, real_type y2,
      real_type x3, real_type y3,
      real_type s0,
      real_type s1,
      int_type  icurve
    ) {
      m_p1[0] = x1; m_p1[1] = y1;
      m_p2[0] = x2; m_p2[1] = y2;
      m_p3[0] = x3; m_p3[1] = y3;
      m_s0     = s0;
      m_s1     = s1;
      m_icurve = icurve;
    }

    int_type  Icurve() const { return m_icurve; }

    real_type x1() const { return m_p1[0]; }
    real_type y1() const { return m_p1[1]; }

    real_type x2() const { return m_p2[0]; }
    real_type y2() const { return m_p2[1]; }

    real_type x3() const { return m_p3[0]; }
    real_type y3() const { return m_p3[1]; }

    real_type S0() const { return m_s0; }
    real_type S1() const { return m_s1; }

    void
    translate( real_type tx, real_type ty ) {
      m_p1[0] += tx; m_p2[0] += tx; m_p3[0] += tx;
      m_p1[1] += ty; m_p2[1] += ty; m_p3[1] += ty;
    }

    void
    rotate( real_type angle, real_type cx, real_type cy );

    void
    scale( real_type sc ) {
      m_p1[0] *= sc; m_p1[1] *= sc;
      m_p2[0] *= sc; m_p2[1] *= sc;
      m_p3[0] *= sc; m_p3[1] *= sc;
    }

    void
    bbox(
      real_type & xmin, real_type & ymin,
      real_type & xmax, real_type & ymax
    ) const {
      minmax3( m_p1[0], m_p2[0], m_p3[0], xmin, xmax );
      minmax3( m_p1[1], m_p2[1], m_p3[1], ymin, ymax );
    }

    real_type baricenterX() const { return (m_p1[0]+m_p2[0]+m_p3[0])/3; }
    real_type baricenterY() const { return (m_p1[1]+m_p2[1]+m_p3[1])/3; }

    real_type const * P1() const { return m_p1; }
    real_type const * P2() const { return m_p2; }
    real_type const * P3() const { return m_p3; }

    bool overlap( Triangle2D const & ) const;

    int_type
    isCounterClockwise() const {
      return G2lib::isCounterClockwise( m_p1, m_p2, m_p3 );
    }

    int_type
    isInside( real_type x, real_type y ) const {
      real_type const pt[2] = {x,y};
      return isPointInTriangle( pt, m_p1, m_p2, m_p3 );
    }

    int_type
    isInside( real_type const pt[2] ) const {
      return isPointInTriangle( pt, m_p1, m_p2, m_p3 );
    }

    real_type
    distMin( real_type x, real_type y ) const;

    real_type
    distMax( real_type x, real_type y ) const;

    void
    info( ostream_type & stream ) const
    { stream << "Triangle2D\n" << *this << '\n'; }

    friend
    ostream_type &
    operator << ( ostream_type & stream, Triangle2D const & c );

  };

}
```

### Quick search

### Table of Contents

- Matlab Interface Manual
- C++ API
- MATLAB API

«
hide menu

menu
sidebar
»

### Navigation

- index
- toc
- Clothoids »
- Program Listing for File Triangle2D.hxx

© Copyright 2021, Enrico Bertolazzi and Marco Frego.
Created using Sphinx 4.2.0.
